# Combined Anti-inflammatory and Neuroprotective Treatments Have the Potential to Impact Disease Phenotypes in *Cln3*^−/−^ Mice

**DOI:** 10.3389/fneur.2019.00963

**Published:** 2019-09-11

**Authors:** Marta A. Tarczyluk-Wells, Christoph Salzlechner, Allison R. Najafi, Ming J. Lim, David Smith, Frances M. Platt, Brenda P. Williams, Jonathan D. Cooper

**Affiliations:** ^1^Department of Basic and Clinical Neuroscience, King's College London, Institute of Psychiatry, Psychology and Neuroscience, Maurice Wohl Clinical Neuroscience Institute, London, United Kingdom; ^2^Department of Pediatrics, David Geffen School of Medicine at UCLA, Los Angeles Biomedical Research Institute at Harbor-UCLA Medical Center, Torrance, CA, United States; ^3^Guy's and St. Thomas' NHS Foundation Trust, King's Health Partners Academic Health Science Centre, Evelina London Children's Hospital, London, United Kingdom; ^4^Faculty of Life Sciences and Medicine, King's College London, London, United Kingdom; ^5^Department of Pharmacology, University of Oxford, Oxford, United Kingdom; ^6^Department of Pediatrics, Washington University School of Medicine, St. Louis, MO, United States

**Keywords:** Batten disease, CLN3 disease, inflammation, ibuprofen, lamotrigine, glial activation, neurodegeneration

## Abstract

Batten disease, or juvenile NCL, is a fatal neurodegenerative disorder that occurs due to mutations in the *CLN3* gene. Because the function of CLN3 remains unclear, experimental therapies for JNCL have largely concentrated upon the targeting of downstream pathomechanisms. Neuron loss is preceded by localized glial activation, and in this proof-of-concept study we have investigated whether targeting this innate immune response with ibuprofen in combination with the neuroprotective agent lamotrigine improves the previously documented beneficial effects of immunosuppressants alone. Drugs were administered daily to symptomatic *Cln3*^−/−^ mice over a 3 month period, starting at 6 months of age, and their impact was assessed using both behavioral and neuropathological outcome measures. During the treatment period, the combination of ibuprofen and lamotrigine significantly improved the performance of *Cln3*^−/−^ mice on the vertical pole test, slowing the disease-associated decline, but had less of an impact upon their rotarod performance. There were also moderate and regionally dependent effects upon astrocyte activation that were most pronounced for ibuprofen alone, but there was no overt effect upon microglial activation. Administering such treatments for longer periods will enable testing for any impact upon the neuron loss that occurs later in disease progression. Given the partial efficacy of these treatments, it will be important to test further drugs of this type in order to find more effective combinations.

## Introduction

The neuronal ceroid lipofuscinoses (NCLs; Batten disease) are a group of fatal, autosomal recessively inherited storage disorders (1). Most forms affect children and young adults, and are collectively the most common cause of childhood dementia. Juvenile NCL (JNCL, or CLN3 disease), is caused by mutations in the *CLN3* gene (2), and is the most common of these disease subtypes. The clinical manifestations of JNCL usually begin with visual impairment between 4 and 7 years of age, declining rapidly to blindness at 5–10 years, proceeded by mental and intellectual deterioration, epilepsy, and motor deficits, ending in premature death at a mean age of 24 years (1).

Because the normal function of the transmembrane CLN3 protein still remains unclear (3), it has not yet been possible to devise an effective mechanistically-based therapy for this disorder. Nevertheless, important clues about the downstream effects of Cln3 deficiency have come from analyzing mouse models of JNCL. Such studies have revealed a range of pathological hallmarks (4), including selective loss of inhibitory interneurons (5), a vulnerability of thalamic relay neurons (5, 6) and deep cerebellar and Purkinje neurons (7), early activation of glia that occurs before neuron loss (5, 8, 9), and the presence of a low-level infiltration of the brain by T-lymphocytes (10, 11). Although disease progression occurs more slowly than in other forms of NCL, Cln3 disease mice exhibit impaired performance on tests of motor ability and coordination early in disease progression (6). Together with pathological landmarks, these tests have been used to judge the efficacy of a variety of small molecule interventions aimed at blocking or reducing the severity of the effects of Cln3 deficiency. These include a series of different glutamate receptor antagonists (12–14), which have shown only moderate efficacy, suggesting that glutamate-mediated excitotoxicity is not central to the mechanisms that operate in Cln3 disease.

There is growing evidence for a range of neuroimmune responses occurring during JNCL pathogenesis (4, 15), and therapeutic strategies to target these events are increasingly being tested (16). Both Cln3 deficient mice (*Cln3*^−/−^) and JNCL patients raise autoantibodies against glutamate decarboxylase (GAD65), and other brain autoantigens (17–19). These can be found in the CSF and within the CNS due to compromised integrity of the blood brain barrier (10). Genetically blocking this autoantibody production, or immunosuppression with mycophenolate mofetil (MMF; CellCept), results in improvements in motor function, decreased neuroinflammation, and partial protection of vulnerable neuron populations in *Cln3*^−/−^ mice (20). A subsequent phase II clinical trial of MMF in JNCL patients showed this drug to be well-tolerated (21), but no evidence for efficacy has been reported.

More recently, the immunomodulatory compounds fingolimod and teriflunomide have also been shown to have significant positive effects upon pathology in both Cln1 and Cln3 deficient mice (11). Similarly, 3 phosphodiesterase-4 (PDE4) inhibitors were found to improve motor function, and attenuate glial activation and lysosomal pathology in a mouse model of Cln3 disease (22), providing further evidence for beneficial effects of an anti-inflammatory and neuroprotective strategy in murine JNCL. Taken together, these data provide evidence that appropriately targeting pathogenically relevant neuroinflammation while potentially reducing neuron loss via neuroprotective agents may be of therapeutic benefit in multiple forms of NCL (16).

Anti-inflammatory and neuroprotective compounds have been tested in a several models of injury and disease (23, 24), including lysosomal storage disorders (25–27). Such compounds do not have an adverse impact on wild type mice (28, 29), but it cannot be ruled out that that prolonged exposure to immunosuppression may have adverse effects in *Cln3*^−/−^ mice. Therefore, in this study, we took the practical approach of investigating whether drugs which are already commonly used by patients with neurological problems, including JNCL, and have a good safety profile in both wild type mice and children would have any beneficial effects in *Cln3*^−/−^ mice. Among these commonly used drugs are anti-inflammatories (i.e., ibuprofen) and anticonvulsants (i.e., lamotrigine), which treat seizures during disease progression. Although anticonvulsants can be neuroprotective by preventing seizures, lamotrigine has also been shown to exert neuroprotective roles in models of injury and disease, likely via mechanisms independent of their anticonvulsant properties. Following ischemic stroke, administration of lamotrigine attenuated hippocampal neuron loss (23), and in an MPTP model of Parkinson disease, lamotrigine was shown to limit neuronal death (24). Therefore, we identified these compounds as good initial candidates for testing whether such commonly used drugs may have therapeutic potential in *Cln3*^−/−^ mice. Since this JNCL mouse model shows early motor deficits and glial activation, but no appreciable neuron loss until late in the disease, we selected behavioral tests and brain inflammation as more sensitive measures to determine the effects of the drugs.

Our data show evidence that combined treatment with ibuprofen and lamotrigine results in improvements of certain motor skills in *Cln3*^−/−^ mice, and moderate effects upon astrocyte activation that were most pronounced for ibuprofen alone, but no overt effect upon microglial activation. These data provide proof of principle for this approach, but it will be important to test further drugs of these types in order to find more effective combinations.

## Materials and Methods

### Mice

In this study male and female C57BL/6J wild type (WT) and homozygous *Cln3*-knockout (*Cln3*^−/−^) mice inbred on the same background were used. *Cln3*^−/−^ mice have a normal lifespan and delayed onset of neurodegeneration, but display early glial activation and motor deficits. To mimic the potential clinical application of treated affected children shortly after diagnosis, drugs were administered to symptomatic mice at 6 months of age. Mice were bred and housed under non-sterile conditions, with food and water available *ad libitum*, and were genotyped as described previously (30). All animal procedures were performed in accordance with the principles of the Basel declaration and the UK Animals (Scientific Procedures) Act 1986, under Home Office Project License PPL 70/7364. This protocol was approved by King's College London's Denmark Hill Campus Committee for Animal Ethics and Welfare.

### Drug Treatments

All drugs were supplemented as a dry mixture to powdered RM1 mouse chow (SDS, UK). Ibuprofen (Sigma) was administered at a dose of 100 mg/kg/day, Lamotrigine (Glaxo-Smith-Kline) at 40 mg/kg/day in powdered chow using glass inkwells as food containers. This allowed us to monitor the amount of food consumed per day, and minimized food loss due to mice digging in these containers. Untreated WT (*n* = 12) and *Cln3*^−/−^ (*n* = 7) mice were fed on powdered chow alone (placebo treated). *Cln3*^−/−^ treatment groups were made up of approximately equal numbers of randomly assigned males and females and received ibuprofen (*n* = 7), or ibuprofen and lamotrigine (*n* = 6). Mice were randomly allocated to treatment groups until all groups were filled, with all treatment groups run in parallel in two separate batches, each containing every treatment group. Mice in different treatment groups were kept in separate cages, but experimenters conducting either behavioral or histological analyses were kept blind to treatment status and genotype, until the study was complete and all data collected. When conducting behavioral testing, the apparatus was cleaned between each mouse to minimize any odors left by previous mice.

### Rotarod Test

An accelerating rotarod (Rotamex-5 Rota Rod, Columbus Instruments, OH, USA) was used to measure the motor skill of mice by assessing their ability to maintain balance on a motor-driven, rotating rod. Due to the repeated, multiple test trials used in our rotarod protocol, motor learning also contributes to the rotarod performance of mice. During the training period, mice were placed on the rotarod starting at zero rpm to 48 rpm in 240 s (0.2 rpm/s acceleration). Mice were trained on the rotarod for three consecutive runs. Following training, mice rested for 1.5 h and then were tested for three test trials each consisting of three consecutive runs, with 15 min of rest between the trials. The average latencies to fall from the rotating rod during the testing periods were calculated for each mouse.

### Vertical Pole Test

This test measures the balance, motor coordination, and vertical orientation capability of mice. It was performed as previously described, with minor modifications (31). The mouse is placed, head downward, on top of a vertical, all-thread plated metal rod (diameter: 1.27 cm; height: 60 cm), and the time until the mouse climbs down to the base of the rod is measured in 5 consecutive trials. Each climbing down trial is terminated after 60 s (to avoid exhaustion). If the mouse falls the score is 60 s. The time to climb down (average of the 5 trials in seconds) were calculated for each mouse.

### Histological Processing

At the end of the 3-month drug treatment, 9-month-old *Cln3*^−/−^ mice were perfusion-fixed with 4% paraformaldehyde (in phosphate-buffered saline (PBS), pH 7.4). Nine-month-old untreated wild type (WT) and *Cln3*^−/−^ mice were also perfusion-fixed. The brains were carefully removed and immersion fixed for at least 24 h in 4% paraformaldehyde, followed by cryoprotection at 4°C in a solution of 30% sucrose in PBS containing 0.05% sodium azide. Subsequently, 40 μm coronal sections were cut on a Leitz 1321 freezing microtome (Microm HM 430; Carl Zeiss Ltd., Cambridge, UK) and stored at 4°C in 96 well plates containing cryoprotectant solution [30% ethylene glycol, 15% sucrose, and 0.05% sodium azide in Tris buffered saline (TBS: 50 mM Tris, 150 mM NaCl, pH 7.6)].

### Quantification of GFAP and CD68 Immunoreactivity

To assess the degree of astrocytic and microglial activation, a 1 in 6 series of 40 μm sections from each brain (*n* = 3, untreated WT mice *n* = 6, and Ibuprofen treated *Cln3*^−/−^ mice *n* = 4) was immunohistochemically stained for the astrocytic marker glial fibrillary acidic protein (GFAP) or the microglial marker CD68. Briefly, sections were incubated in 1% H_2_O_2_ in TBS for 30 min to quench endogenous peroxidase activity and rinsed three times in TBS. Sections were then blocked in 15% normal serum (from the host species of the secondary antibody) in TBS-T (TBS containing 0.3% w/v Triton X-100) for 30 min. Sections were then incubated overnight at 4°C with either a rabbit anti-GFAP (1:8,000, Dako) or a rat anti-CD68 (1:2,000, AbD Serotec) diluted in TBS-T containing 10% appropriate normal serum. After rinsing, sections were incubated for 2 h at room temperature with the appropriate biotinylated secondary antibody (for GFAP: swine anti-rabbit, 1:1,000, Dako; for CD68: rabbit anti-rat, 1:1,000, Vector Laboratories) diluted in TBS-T containing 10% normal serum. After rinsing, sections were incubated for 2 h at room temperature in ABC reagent diluted 1:1,000 in TBS (Vectastatin Elite ABC kit, Vector Laboratories). After rinsing, sections were incubated in 0.05% DAB (3,3′-diaminobenzidine; Sigma-Aldrich, Dorset, UK) and 0.001% H_2_O_2_ in TBS to visualize immunoreactivity. Afterwards sections were mounted on Superfrost microscope slides, air dried overnight, cleared in xylene and coverslipped with DPX (VWR).

Sections stained for either GFAP or CD68 were scanned on a Zeiss AxioImager.M2 using a x20 objective and brightfield illumination using *StereoInvestigator* (MBF Biosciences, Williston, VT). Images were exported as TIFF files and imported into *Image Pro Premier* (Media Cybernetics, Rockville, MD) and the relative proportion of each structure positive for each antigen was determined using smart segmentation to apply a threshold that discriminated specific immunoreactivity from background staining.

### Statistical Analysis

Statistical analysis of all behavioral data was done using repeated measures two-way ANOVAs with Bonferroni's test using *GraphPad Prism* 7. Histological data were analyzed by one-way ANOVA with Bonferroni's post-test. Results were considered statistically significant when *P* < 0.05. Our sample sizes were calculated using *PS Power Sample Size* software to provide sufficient power (95%) to detect a 10% difference between the means of the groups, which was considered the minimum indication of efficacy.

## Results

### Moderate Impact of Drug Treatments Upon Rotarod and Vertical Pole Testing Performance

**Figure 1:**
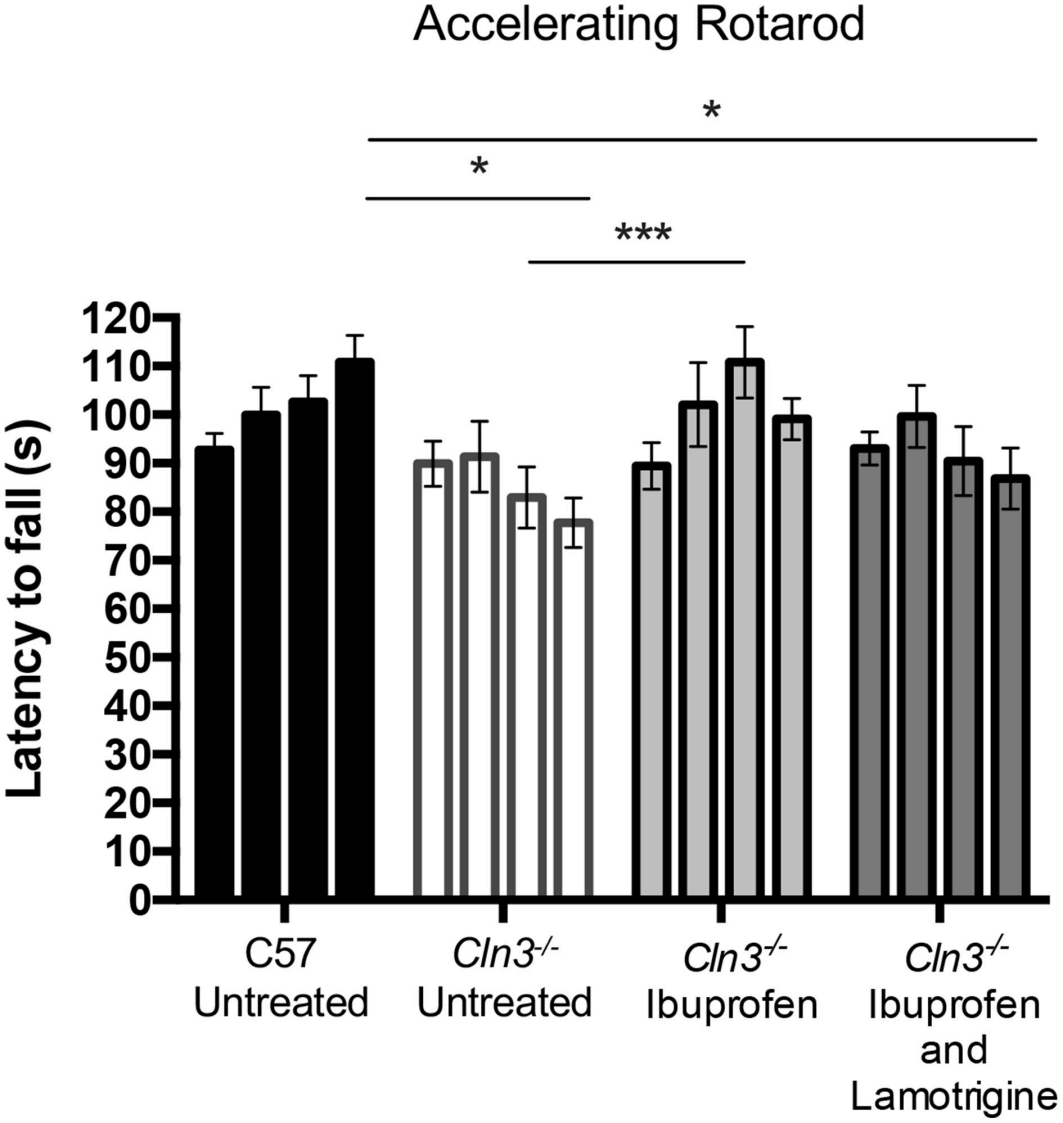
A 3-month treatment with Ibuprofen induces moderate improvement of motor skills in 6–9-month old *Cln3*^−/−^ mice. An accelerating rotarod (from 0 to 24 rpm in 240 s) was used to measure the motor skills of 6–9-month-old *Cln3*^−/−^ and wild type (WT) (*n* = 12) mice. Mice were given either powdered diet with Ibuprofen, Ibuprofen and Lamotrigine, or powdered diet with no drugs (Untreated) (*n* = 6–7) for 3 months. Data bars represent mean ± S.E.M. of the time (s) mice were able to stay on the rotating rod at 6–9 months of age (0–3 months of treatment). Repeated measures two-way ANOVA was applied with Bonferroni's post-test (^*^p < 0.05 and ^***^p < 0.001).

**Figure 2:**
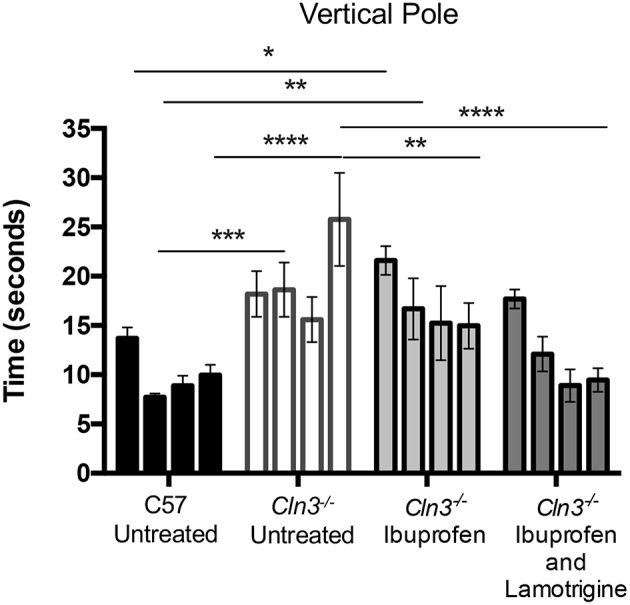
A 3-month treatment with combination of Ibuprofen and Lamotrigine induces significant improvement of motor skills in 6–9-month old *Cln3*^−/−^ mice. A vertical pole was used to measure the motor skills of 6–9-month-old WT (*n* = 12) mice and *Cln3*^−/−^ mice given diet with Ibuprofen, Ibuprofen and Lamotrigine, or powdered diet with no drugs (Untreated) (*n* = 6–7). Data points represent mean ± S.E.M. of the time (s) mice took to climb down a vertical pole at 6–9 months of age (0–3 months of treatment). Repeated measures two-way ANOVA was applied to compare Control and drug treated mice (^*^p < 0.05, ^**^p < 0.01, ^***^p < 0.001, and ^****^p < 0.0001).

Like mouse models of other forms of NCL, Cln3 deficient mice show a progressive decline in their performance upon the accelerating rotarod (7), which is detectable relatively early in disease progression depending on the precise protocol used. The latency to fall from the rotarod is a commonly used readout of efficacy for potential therapeutic compounds in these mice (20). Another test of motor performance is the time taken to descend from the top of a vertical metal pole, a task in which *Cln3*^−/−^ mice perform progressively worse with age (31). We used both tests as outcome measures to compare the impact in *Cln3*^−/−^ mice of chow-administered ibuprofen (100 mg/kg/day), either given alone or in combination with lamotrigine (40 mg/kg/day). Since ibuprofen and lamotrigine (alone and in combination) have previously been studied in wild type (WT) mice, and data from our group (not shown here) also showed no effect of these drugs on healthy mice, the treated *Cln3*^−/−^ mice were only compared to untreated/placebo *Cln3*^−/−^ or wild type (WT) control mice. These treatments began when the mice were 6 months of age and continued for 3 months, with rotarod performance (Figure 1) and vertical pole test performance (Figure 2) tested at monthly intervals.

Over the treatment period, untreated WT mice were better able to stay on the accelerating rotarod, whereas the performance of untreated *Cln3*^−/−^ mice was worse, resulting in a significantly shorter latency to fall at 9 months of age. In contrast, *Cln3*^−/−^ mice treated with ibuprofen displayed a progressively longer latency to fall, a performance that was significantly better than untreated *Cln3*^−/−^ mice after 2 months of treatment. Combined treatment with ibuprofen and lamotrigine did not produce the same beneficial effects, although the rotarod performance of these combination treated *Cln3*^−/−^ mice was not as impaired as in untreated mutants.

Downward facing vertical pole testing also revealed that with repeated testing, untreated WT mice took less time to descend the vertical pole, whereas untreated *Cln3*^−/−^ mice consistently took longer to achieve this task and displayed a significantly worse performance by 9 months of age (Figure 2). Combined treatment with ibuprofen and lamotrigine shortened the time that *Cln3*^−/−^ mice took to descend the vertical pole, with this effect was significant at 9 months of age. In comparison, ibuprofen treatment alone also improved performance in the downward facing pole test, but not to the same extent as combined treatment. Nevertheless, despite being less effective than combination therapy, this treatment effect of ibuprofen alone was also significant at 9 months of age compared to untreated *Cln3*^−/−^ mice.

### Cell-Type Specific Impact of Drug Treatments Upon Glial Activation in Cln3 Mice

Compared to earlier onset types of NCL, *Cln3*^−/−^ mice display a relatively slowly progressing neurodegenerative phenotype (5, 8), and neuron loss does not typically become significant until later in disease progression. However, this neuron loss is characteristically preceded by a low-level glial activation with both astrocyte and microglial activation occurring in brain regions where neuron loss is subsequently most pronounced (5, 8). In particular, this includes the thalamic nuclei that relay somatosensory (ventral posterior, VPM/VPL) and visual information (dorsolateral geniculate, DLG) to the corresponding regions of somatosensory barrelfield (S1BF) and primary visual (V1) cortex, respectively. Immunostaining for glial fibrillary associated protein (GFAP, astrocyte reactivity) and CD68 (microglial activation) in these nuclei have been used as pathological readouts in numerous studies in Cln3-deficient mice (20, 22, 32). We examined these markers in the VPM/VPL, S1BF, DLG, and V1 of untreated WT and *Cln3*^−/−^ mice, and mutant mice treated with ibuprofen and the combination of ibuprofen and lamotrigine.

**Figure 3:**
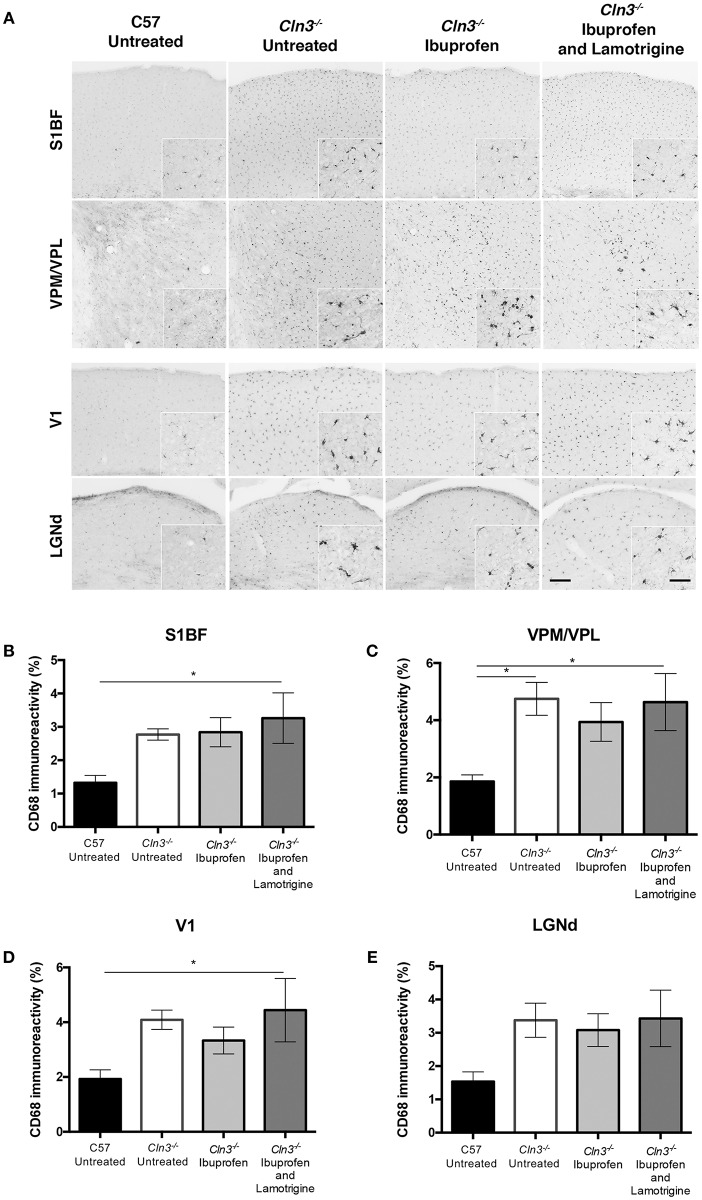
Effect of Ibuprofen and Lamotrigine treatment on microglial reactivity in 9-month old *Cln3*^−/−^ mice. **(A)** Representative images of CD68 immunostaining in S1BF, VPM/VPL, V1, and LGNd. Scale bars, 200 and 25μm (inserts). **(B–E)** Quantification of % CD68 immunoreactivity in S1BF, VPM/VPL, V1, and LGNd, respectively. Data points represent mean ± S.E.M. of the % CD68 immunoreactivity. Ordinary one-way ANOVA was applied with Bonferroni's post-test (^*^p < 0.05).

Immunostaining for CD68 revealed the typical low level of microglial activation that was evident in *Cln3*^−/−^ mice, with somewhat more darkly stained microglia evident in both thalamic relay nuclei and cortical regions (Figure 3). As reported previously (9), these CD68-positive microglia in untreated *Cln3*^−/−^ mice exhibited a moderately larger cell soma, but did not display the hypertrophied morphology typical of brain macrophages. CD68-stained microglia in *Cln3*^−/−^ mice treated with either ibuprofen alone or in combination with lamotrigine appeared very similar in terms of both staining intensity and morphology, suggesting very little impact of these drug treatments upon microglial activation. Thresholding image analysis of the level of CD68 immunostaining confirmed these morphological observations, and although ibuprofen treatment alone appeared to result in marginally less CD68 staining, none of these treatment effects approached statistical significance.

**Figure 4:**
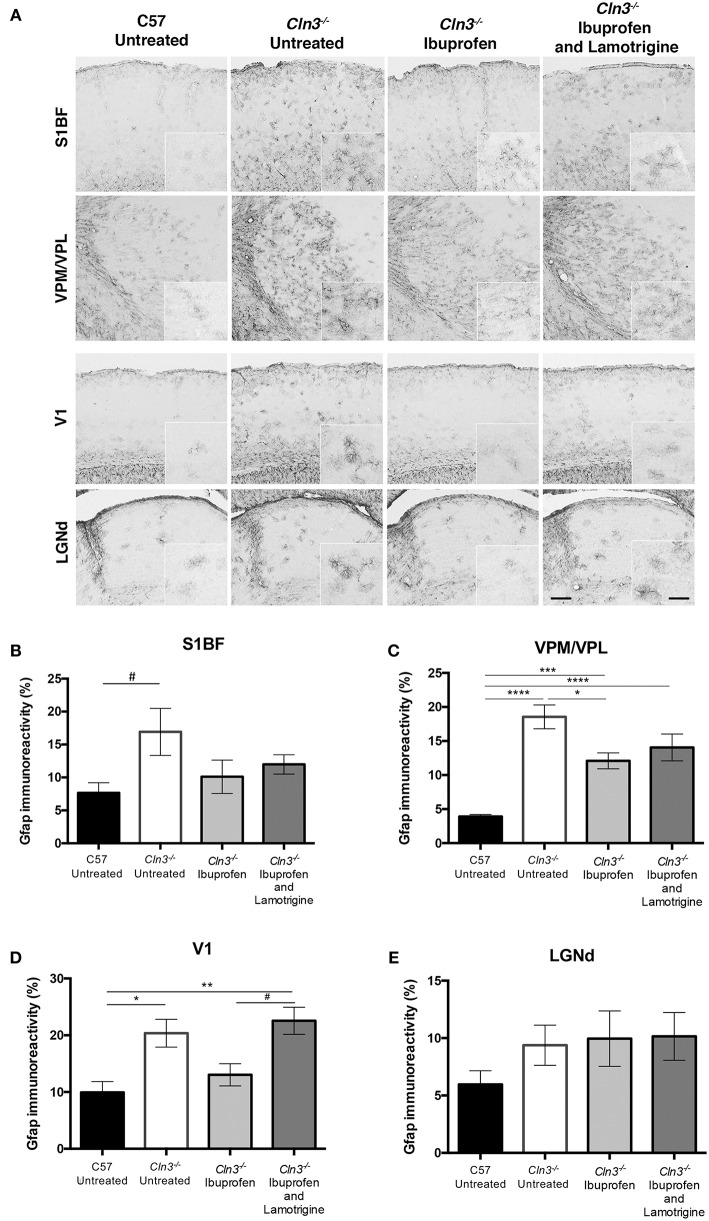
Moderate effect of Ibuprofen and Lamotrigine treatment on JNCL astrocyte immunoreactivity in 9-month old *Cln3*^−/−^ mice. **(A)** Representative images of GFAP immunostaining in S1BF, VPM/VPL, V1, and LGNd. Scale bars, 200 and 25μm (inserts). **(B–E)** Quantification of % GFAP immunoreactivity in S1BF, VPM/VPL, V1, and LGNd, respectively. Data points represent mean ± S.E.M. of the % GFAP immunoreactivity. Ordinary one-way ANOVA was applied with Bonferroni's post-test (^*^p < 0.05, ^**^p < 0.01, ^***^p < 0.001, ^****^p < 0.0001, and ^#^1 < *p* > 0.05).

Consistent with previous reports (5, 8), immunostaining for the astrocyte marker GFAP revealed more intense and widespread staining in untreated *Cln3*^−/−^ mice than in untreated WT mice in both thalamic relay nuclei and cortical regions (Figure 4). Although astrocytes in untreated *Cln3*^−/−^ mice appeared more intensely stained, they exhibited the morphology of being only partly activated, as previously reported (9). In contrast, in *Cln3*^−/−^ mice treated with ibuprofen alone there qualitatively appeared to be fewer and less intensely stained GFAP-positive astrocytes throughout the cortex than in untreated mutants, but still more than were evident in untreated WT mice. In combination-treated *Cln3*^−/−^ mice, the distribution of GFAP immunoreactivity in the thalamus and across the cortex more closely resembled that in untreated *Cln3*^−/−^ mice, suggesting there was less of an impact of combined treatment upon astrocyte activation than ibuprofen alone. Quantifying these changes in GFAP immunoreactivity via thresholding image analysis in somatosensory and visual pathways largely confirmed these observations, and revealed that despite qualitative trends in the data, the effect of ibuprofen treatment upon astrogliosis was only significant within the VPM/VPL (Figures 4B–E).

Taken together these data reveal evidence for a modest and regionally-dependent efficacy of ibuprofen treatment against astrogliosis, but not upon microglial activation, in *Cln3*^−/−^ mice. In contrast to its moderately positive effects upon rotarod and vertical pole testing, the combined treatment with ibuprofen and lamotrigine had no additional impact upon attenuation of glial activation in *Cln3*^−/−^ mice.

## Discussion

There have been a series of different therapeutic interventions that have targeted specific parts of the neuroimmune response in JNCL (16). One of these approaches used the immunosuppressant MMF in Cln3-deficient mice (20). Although the impact of MMF was broadly positive upon the CLN3 disease phenotype of these mice, prolonged exposure to MMF was associated with a decline in rotarod performance (20). In order to find a strategy that avoided such adverse effects of immunosuppression, we conducted a proof of concept study to test whether a cheap and widely available anti-inflammatory drug that is already approved for use in children would have any therapeutic benefit in *Cln3*^−/−^ mice, when given alone or in combination with lamotrigine. These compounds have previously been tested extensively in wild type mice (28, 29), and in other similar lysosomal storage disorders (25–27). Additionally, previous data from our group (not shown here), showed that neither of the drugs alone or in combination had any effect on wild type mice. Therefore, we adopted a simple study design directly comparing treated and untreated *Cln3*^−/−^ mice to wild type controls. Our data provide proof of concept that such drugs, which are approved for use in children for other indications, are capable of partly influencing both behavioral and certain pathological phenotypes of these Cln3-deficient mice. These effects are relatively modest, and this may reflect that we started our treatment in mice that were beginning to show the behavioral effects of disease, rather than being completely pre-symptomatic. Indeed, it is plausible that such treatments may provide additional benefit if given earlier in disease progression, or together with other therapies. It will be important to systematically test other compounds of this type to find more effective drug combinations, as has been done in other lysosomal storage disorders (25–27).

The discovery that mutations in the *CLN3* gene cause JNCL was made nearly 25 years ago (2), but the precise mechanisms that cause the devastating impact upon affected individuals remain unclear. Most experimental therapies for this disorder have largely focused on blocking the downstream effects of *CLN3* mutation (1, 33). This includes the variety of neuroimmune responses that occur early in disease progression (5, 8), which now appear to contribute to JNCL pathogenesis. Infiltration of peripheral immune cells into the CNS in JNCL is relatively minor (10, 11), but such responses may still influence disease progression (34). Genetic or pharmacological strategies that block this infiltration provide some degree of therapeutic benefit in Cln3-deficient mice (11, 20). Similar strategies have also been tested in infantile NCL by crossing to Rag1-deficient mice (34), or immunomodulatory drug treatment (11), both positively influencing disease progression in both Ppt1 and Cln3-deficient mice. This suggests that an adaptive immune response plays at least some part in influencing disease progression in multiple forms of NCL.

There is a close relationship between where glial activation occurs and the extent of subsequent neuron loss in multiple forms of NCL (4, 15). There is now mounting evidence that this innate immune response in JNCL may contribute to neuronal dysfunction and loss, with Cln3 deficiency impacting the function of both astrocytes and microglia (9, 35, 36). Furthermore, in co-culture systems the presence of Cln3-deficient astrocytes and microglia harms WT neurons, and promotes the death of Cln3-deficient neurons (9). While the mechanisms that underlie these events remain unclear, strategies that suppress or modulate an innate response appear to have promise. For example, PDE-4 inhibitors provided partial attenuation of glial activation and behavioral benefits in addition to neuroprotective effects (22).

Compared to the partial success of these targeted interventions, the efficacy of our approach to repurpose commonly used anti-inflammatory drugs is more modest. As already discussed, starting drug administration earlier in disease progression may be more effective, or other non-steroidal or other anti-inflammatory drugs may prove more effective. It will also be important to test such drugs over a longer time period when neuron loss becomes evident in this mouse model (5), to determine if they are capable of neuroprotective effects. Staining for additional markers of glial activation is likely to be informative at these more advanced stages of disease progression, but as we have reported previously microglial activation in Cln3 deficient mice remains at a relatively low level, even at disease end stage (9). With no direct evidence for astrocyte proliferation in Cln3 deficient mice (5, 8), it appears these effects are exerted upon the relative level of astrocyte activation. A consistent feature of mouse models of NCL is the relative vulnerability of the thalamus compared to the cortex [reviewed in Cooper et al. (4)] and this includes mouse models of CLN3 disease (5, 6). The reasons for this apparent vulnerability are unclear, but we and others have used proteomic analysis to gain insights into this issue in other forms of NCL (37–39), revealing concurrent and early alterations in the expression of a variety of neuronal and inflammatory markers in the thalamus. These are consistent with the notion of the thalamus as a focus for disease in multiple forms of NCL, and further emphasize a potential role for neuroimmune responses in their pathogenesis.

Nevertheless, regardless of the underlying mechanisms, the fact that even relatively short term administration of a drug such as ibuprofen that has an established safety profile, had any impact at all upon behavior or neuropathological changes is still encouraging. We are currently testing more drugs of this type to identify more effective combinations, particularly with regards to microglial activation. It will also be important to discern the exact inflammatory profile of wild type and mutant mice treated with neuroprotectants and anti-inflammatories in future studies, as morphological and histological changes of astrocytes and microglia could be indicative of a range of activation states. Notably, activation of microglia may indeed favor a neuroprotective role for anti-inflammatory drugs (40). Additionally, increasing expression of neuroprotective agents was shown to exert anti-inflammatory effects in INCL mice, despite having no effect on astrocyte or microglial reactive morphology (41). Although these immunomodulatory approaches have shown some efficacy (11, 16), they may only be targeting more secondary downstream consequences of Cln3 deficiency and are unlikely to be curative. Gene therapy is the likeliest strategy to succeed in JNCL and has now been shown to have some promise in JNCL mice (32). Nevertheless, there still remains scope for neuromodulatory or anti-inflammatory approaches to be given either before gene therapy can be administered or as an adjunct to this approach, as we have done in Cln1 disease mice (42). In this respect drugs which are inexpensive and have comparative anti-inflammatory effects, but less immunosuppressive side effects, and an established safety profile in children may prove to be of clinical utility, if we can identify more effective drugs or drug combinations of this type.

## Data Availability

The datasets generated for this study are available on request to the corresponding author.

## Ethics Statement

All animal procedures were performed in accordance with the UK Animals (Scientific Procedures) Act 1986, under Home Office Project Licence PPL 70/7364. This protocol was approved by King's College London's Denmark Hill Campus Committee for Animal Ethics and Welfare.

## Author Contributions

MT-W: contributed to study concept and design, performed all of the histology, set up drug treatments and behavioral testing and ran most of the behavioral testing, and drafted manuscript. CS: did all of the mouse genotyping and some of the drug treatments and behavioral testing, under the supervision of MT-W. AN: drafted manuscript, analyzed much of the histology, and formatted figures. ML: clinical advisor on study design and manuscript editing. DS: contributed to study design (drug treatment and behavioral testing). FP and BW: contributed to study concept and design, data interpretation, and manuscript editing. JC: Principal Investigator for the project, contributed to study concept and design, data interpretation, and manuscript editing.

### Conflict of Interest Statement

The authors declare that the research was conducted in the absence of any commercial or financial relationships that could be construed as a potential conflict of interest.
